# The distinctive cell division interactome of *Neisseria gonorrhoeae*

**DOI:** 10.1186/s12866-017-1140-1

**Published:** 2017-12-12

**Authors:** Yinan Zou, Yan Li, Jo-Anne R. Dillon

**Affiliations:** 1 Department of Microbiology and Immunology, College of Medicine, Saskatoon, SK S7N 5E5 Canada; 2 Vaccine and Infectious Disease Organization, International Vaccine Centre, Saskatoon, SK S7N 5E3 Canada; 3 Department of Biology, College of Arts and Science, University of Saskatchewan, Saskatoon, SK S7N 5A5 Canada

**Keywords:** Cell division, Interactome, *N. gonorrhoeae*, Protein-protein interaction, Bacterial two-hybrid assay, Surface plasmon resonance, GST pull-down, FtsA domains

## Abstract

**Background:**

Bacterial cell division is an essential process driven by the formation of a Z-ring structure, as a cytoskeletal scaffold at the mid-cell, followed by the recruitment of various proteins which form the divisome. The cell division interactome reflects the complement of different interactions between all divisome proteins. To date, only two cell division interactomes have been characterized, in *Escherichia coli* and in *Streptococcus pneumoniae*. The cell divison proteins encoded by *Neisseria gonorrhoeae* include FtsZ, FtsA, ZipA, FtsK, FtsQ, FtsI, FtsW, and FtsN. The purpose of the present study was to characterize the cell division interactome of *N. gonorrhoeae* using several different methods to identify protein-protein interactions. We also characterized the specific subdomains of FtsA implicated in interactions with FtsZ, FtsQ, FtsN and FtsW.

**Results:**

Using a combination of bacterial two-hybrid (B2H), glutathione S-transferase (GST) pull-down assays, and surface plasmon resonance (SPR), nine interactions were observed among the eight gonococcal cell division proteins tested. ZipA did not interact with any other cell division proteins. Comparisons of the *N. gonorrhoeae* cell division interactome with the published interactomes from *E. coli* and *S. pneumoniae* indicated that FtsA-FtsZ and FtsZ-FtsK interactions were common to all three species. FtsA-FtsW and FtsK-FtsN interactions were only present in *N. gonorrhoeae*. The 2A and 2B subdomains of FtsA_Ng_ were involved in interactions with FtsQ, FtsZ, and FtsN, and the 2A subdomain was involved in interaction with FtsW.

**Conclusions:**

Results from this research indicate that *N. gonorrhoeae* has a distinctive cell division interactome as compared with other microorganisms.

**Electronic supplementary material:**

The online version of this article (10.1186/s12866-017-1140-1) contains supplementary material, which is available to authorized users.

## Background

Cell division is essential for bacterial survival. In *Escherichia coli* (Ec), normal cell division is driven by the formation of an FtsZ-ring at the division site [1], followed by the recruitment of other essential proteins, which together form the divisome [2]. Genes encoding most cell division proteins are located in a conserved region, the *d*ivision and *c*ell *w*all (*dcw*) cluster [3]. *dcw* clusters have been identified in most bacterial species, including *E. coli*, *Bacillus subtilis* (Bs), *Streptococcus pneumoniae* (Sp), *Caulobacter crescentus* (Cc) and *Neisseria gonorrhoeae* (Ng) [4–7]. Although the gene organization of the *dcw* cluster varies in different bacteria species [8], proteins involved in the cell division process are relatively conserved [9, 10].

*E. coli* encodes ten essential cell division proteins, including FtsZ, FtsA, ZipA, FtsK, FtsQ, FtsB, FtsL, FtsW, FtsI, and FtsN [11, 12]. Assembly of the FtsZ-ring structure is initiated with the polymerization of FtsZ, driven by GTP hydrolysis, at the mid-cell [13]. FtsA and ZipA are recruited by FtsZ and anchor FtsZ to the inner membrane [14]. After the recruitment of FtsK, a DNA translocase involved in DNA segregation [15–17], the protein complexes FtsQ-FtsB-FtsL and FtsW-FtsI are localized to the septal ring, sequentially [15, 18]. Recent studies showed that the FtsQ-FtsB-FtsL complex serves as a signal sensor which promotes cell wall remodeling necessary for cell constriction [19]. FtsI is a high-molecular-weight transpeptidase that cross-links glycan strands. The FtsW-FtsI complex is part of the peptidoglycan synthesis machinery, and FtsW, a lipid II flippase, transports the cell wall precursor across the membrane [20, 21]. FtsN is recruited as the last essential division protein that initiates cell constriction [22].

Using a bacterial two-hybrid (B2H) assay, an *E. coli* cell division protein-protein interaction network, the cell division interactome, which included 16 interactions between 10 cell divison proteins, was identified [23, 24]. The cell division interactome of *S. pneumoniae* was also characterized using a combination of B2H and co-immunoprecipitation assays [25]. A total of 17 interactions was observed among nine cell division proteins of *S. pneumoniae* which included FtsZ, FtsA, FtsK, DivlB, DivlC, FtsL, FtsW, and PBP2x [25]. To date, *E. coli* and *S. pneumoniae* are the only two organisms with characterized cell division interactomes [23–25].

*N. gonorrhoeae* is a Gram-negative diplococcus that causes gonorrhea in humans [26]. Previous studies on *N. gonorrhoeae* cell division focused on its Min system which localizes FtsZ to the mid-cell, and FtsZ [27–29]. *N. gonorrhoeae* also contains a *dcw* cluster which encodes 5 cell division proteins - FtsZ, FtsA, FtsQ, FtsW, and FtsI [7]. Other non-*dcw* cluster divisome proteins encoded by *N. gonorrhoeae* include ZipA, FtsK, and FtsN. As compared to *E. coli*, *N. gonorrhoeae* lacks FtsB and FtsL [7].

To investigate the cell division interactome in *N. gonorrhoeae*, its cell division protein interactions were identified using a combination of B2H and glutathione S-transferase (GST) pull-down assays, as well as surface plasmon resonance (SPR). We identified nine interactions among the eight cell division proteins tested. We also identified the subdomains of FtsA_Ng_ involved in its interaction with FtsQ_Ng_, FtsZ_Ng_, FtsN_Ng_, and FtsW_Ng_. Comparison of the cell division interactomes of *E. coli*, *S. pneumoniae* and *N. gonorrhoeae* indicates that *N. gonorrhoeae* possesses a distinctive cell division interactome.

## Methods

### Strains and growth conditions

The bacterial strains and plasmids used in this study are shown in Table 1. *E. coli* DH5α and XL1-Blue were used as hosts for cloning. *E. coli* BL21 (DE3) and C41 (DE3) were used as hosts for protein purification. *E. coli* R721 was used in B2H assays [30]. *E. coli* DH5α, XL1-Blue, BL21(DE3) and C41 (DE3) were grown in Luria-Bertani (LB) medium (BD Difco™, Sparks, MD), for 16–18 h (hr), at 37 °C. *E. coli* R721 was grown under the same conditions and incubated at 34 °C, as described previously [24].

**Table 1:** Bacterial strains used in this study

Stain	Relevant characteristics	Source/reference
*E. coli* DH5α	*supE44 ΔlacU169* (*80lacZΔM15*) *hsdR17 endA1 gyrA96 thi-1 relA1*	Gibco
*E. coli* XL1-Blue	*recA1 endA1 gyrA96 thi-1 hsdR17 supE44 relA1 lac* [*F′ proAB laclq ZΔM15*] Tn10	Stratagene
*E. coli* BL21(DE3)	F^−^ *, dcm Δ, ompT, hsdS* (r^−^_B_ m^−^_B_)*, gal, λ(DE3)*	Stratagene
*E. coli* C41 (DE3)	F^−^ *ompT hsdSB* (r^−^_B_ m^−^_B_) *gal dcm (srl-recA)* 306::Tn10 (Tet^r^) (*DE3*)	[70]
*E. coli* R721	*supE thy D*(*lac-proAB*) F′ [*proAB* ^*+*^ *lacI* ^*q*^ *lac*ZDM15] *glpT*::O-_P434/P22_ *lacZ*	[30]
*N. gonorrhoeae* CH811	Auxotype (A)/serotype (S)/plasmid content (P) class: nonrequiring/IB-2/plasmid-free, Str^r^	[71]

*N. gonorrhoeae* CH811 was grown on GC medium base agar (GCMB, Oakville, ON), supplemented with Kellogg’s defined supplement (GCMBK, 40 g D-glucose, 1 g glutamine, 10 ml of 0.5% ferric nitrate and 1 ml of 20% cocarboxylase), at 35 °C, in a humid environment, with 5% CO_2_, for 18 to 24 h [31].

When required, the following concentrations of antibiotics were added to LB medium: 100 μg/ml ampicillin (Sigma, Oakville, ON) or 50 μg/ml kanamycin (Sigma). For B2H assays, 34 μg/ml chloramphenicol (Sigma), 30 μg/ml kanamycin, and 50 μg/ml ampicillin were added to LB medium.

### DNA manipulations

*N. gonorrhoeae* CH811 genomic DNA was purified using a QIAamp® genomic DNA kit (Qiagen, Mississauga, Ontario, Canada). DNA samples were stored at −20 °C. Oligonucleotides for polymerase chain reaction (PCR) amplifications were synthesized by Invitrogen (Table 2; Burlington, Ontario, Canada). PCRs were performed in a GeneAmp® PCR system 9700 (Applied Biosystems, Foster City, CA, USA) as follows: 4 min (min) at 94 °C, 30 cycles of denaturation for 1 min at 94 °C, annealing for 45 s (s) at 55 °C, extension for 1.5 mins at 72 °C, and 10 mins at 72 °C. PCRs were carried out in 100-μl (final volume) mixtures comprising 71.5 μl double-distilled H_2_O (ddH_2_O), 10 μl of 10× PCR buffer [15 mM MgCl_2_, 4 μl of 10 mM deoxynucleoside triphosphate (dNTP), 2 μl of each primer (0.2 μg/ml), 0.5 μl of Taq DNA polymerase (5 U/μl; New England BioLabs, Ontario, Canada)], and, 10 μl of purified *N. gonorrhoeae* CH811 genomic DNA suspension.

**Table 2:** Primers designed in this study

Primer name		Sequences (5′-3′)
P1	FtsA-reBamHI	GCGCGGATCCTCAGAGGTTGTTTTCAATCC
P2	FtsA-fwSalI	GCGCGTCGACCATGGAACAGCAGAAAAGATAC
P3	fwSalI-ftsK	GCGCGTCGACCATGTTTTGGATAGTTTTGATCGTTAT
P4	reBamHI-ftsK	CGCGGGATCCTCAAGCATTGTCCAAGGGGACGAG
P5	fwSalI-ftsQ	GCGCGTCGACCATGTGGGATAATGCCGAAGCGATG
P6	reBamHI-ftsQ	CGCGGGATCCCTATTCTTCGGATTCTTTTTCGGG
P7	fwSalI-ftsI	GCGCGTCGACCATGTTGATTAAAAGCGAATATAAGCC
P8	reBamHI-ftsI	CGCGGGATCCTTAAGACGGTGTTTTGACGGCTGC
P9	fwSalI-ftsW	GCGCGTCGACCATGAAGATTTCGGAAGTATTGGTAAA
P10	reBamHI-ftsW	CGCGGGATCCTTACTCCACCCGGTAACCGCGCAT
P11	fwSalI-ftsN	GCGCGTCGACCATGTTTATGAACAAATTTTCCCAATC
P12	reBamHI-ftsN	CGCGGGATCCTTATTTGCCTTCAATCGCACGGAT
P13	fwBglII-ZipA	GCGCGAGATCTGATGATTTACATCGTACTGTTCCTC
P14	reBamHI-ZipA	CGCGGGATCCTTATGAAAACAGGCGCAGGGC
P15	FtsA-reEcoRI-pET30a	ATATCGAATTCTCAGAGGTTGTTTTCAATCCACC
P16	FtsA-fwBglII-pET30a	AGCCCAGATCTGATGGAACAGCAGAAAAGATACATC
P17	fwBglII-FtsQ- pET30a	AGCCCAGATCTGATGTGGGATAATGCCGAAGCGATG
P18	reEcoRI-ftsQ- pET30a	ATATCGAATTCCTATTCTTCGGATTCTTTTTCGGG
P19	FtsZ-fwBgl II-pET30a	AGCCCAGATCTGATGGAATTTGTTTACGACGTGGCA
P20	FtsZ-ReEcoRI-pET30a	AGCCCGAATTCTTATTTGTCTGAATTGTGTTGACG
P21	fwFtsA-BamHI-GST	CGCGGGATCCATGGAACAGCAGAAAAGATACATC
P22	fwEcoRI-FtsN	GACGAATTCATGTTTATGAACAAATTTTCCCAATCC
P23	reXhoI-FtsN	GACCTCGAGTTATTTGCCTTCAATCGCACG

### Bacterial two-hybrid assays

The method developed by Di Lallo et al. [24] was used for all B2H assays. *ftsA*, *ftsK*, *ftsQ*, *ftsI*, *ftsW*, and *ftsN* were amplified from *N. gonorrhoeae* CH811 by PCR using the primer pairs P1/P2, P3/P4, P5/P6, P7/P8, P9/P10, and P11/P12 (Table 2), respectively. PCR amplicons were digested with BamHI and SalI and ligated into previously digested pcI_p22_ and pcI_434_ vectors, to produce pcl_p22_-A, pcI_p22_-K, pcI_p22_-I, pcI_p22_-W, pcI_p22_-Q, pcI_p22_-N, pcI_434_-A, pcI_434_-K, pcI_434_-I, pcI_434_-W, pcI_434_-Q, and pcI_434_-N (Table 3). *zipA*_Ng_ was amplified from *N. gonorrhoeae* CH811 genomic DNA using the primer pair P13/P14 (Table 2); the PCR amplicons was digested with BglII and BamHI, and ligated into pre-digested pcI_p22_ and pcI_434_ to produce pcI_p22_-ZipA and pcI_434_-ZipA. pcI_p22_-Z and pcI_434_-Z constructs were generated previously [32].

**Table 3:** Plasmids used in this study

Plasmid	Relevant genotype	Source/Reference
pcI_p22_	pC132 derivative carrying N-terminal end of P22 repressor	[30]
pcI_434_	pACYC177 derivative carrying N-terminal end of 434 repressor	[30]
pcI_p22_-A	pcIp_22_ derivative carrying the *ftsA*_*Ng*_ gene	This study
pcI_434_-A	pcI_434_ derivative carrying the *ftsA*_*Ng*_ gene	This study
pcI_p22_-K	pcIp_22_ derivative carrying the *ftsK*_*Ng*_ gene	This study
pcI_434_-K	pcI_434_ derivative carrying the *ftsK*_*Ng*_ gene	This study
pcI_p22_-Q	pcIp_22_ derivative carrying the *ftsQ*_*Ng*_ gene	This study
pcI_434_-Q	pcI_434_ derivative carrying the *ftsQ*_*Ng*_ gene	This study
pcI_p22_-I	pcIp_22_ derivative carrying the *ftsI*_*Ng*_ gene	This study
pcI_434_-I	pcI_434_ derivative carrying the *ftsI*_*Ng*_ gene	This study
pcI_p22_-W	pcIp_22_ derivative carrying the *ftsW*_*Ng*_ gene	This study
pcI_434_-W	pcI_434_ derivative carrying the *ftsW*_*Ng*_ gene	This study
pcI_p22_-N	pcIp_22_ derivative carrying the *ftsN*_*Ng*_ gene	This study
pcI_434_-N	pcI_434_ derivative carrying the *ftsN*_*Ng*_ gene	This study
pcI_p22_-Z	pcIp_22_ derivative carrying the *ftsZ*_*Ng*_ gene	[32]
pcI_434_-Z	pcI_434_ derivative carrying the *ftsZ*_*Ng*_ gene	[32]
pcI_p22_-AT1	pcIp_22_ derivative carrying the *ftsA*_*Ng*_ gene fragment encoding amino acids 1–162	[33]
pcI_p22_-AT2	pcIp_22_ derivative carrying the *ftsA*_*Ng*_ gene fragment encoding amino acids 1–194	[33]
pcI_p22_-AT3	pcIp_22_ derivative carrying the *ftsA*_*Ng*_ gene fragment encoding amino acids 1–230	[33]
pcI_p22_-AT4	pcIp_22_ derivative carrying the *ftsA*_*Ng*_ gene fragment encoding amino acids 231–301	[33]
pcI_p22_-AT5	pcIp_22_ derivative carrying the *ftsA*_*Ng*_ gene fragment encoding amino acids 302–414	[33]
pcI_p22_-AT6	pcIp_22_ derivative carrying the *ftsA*_*Ng*_ gene fragment encoding amino acids 351–414	[33]
pcI_434_-AT1	pcI_434_ derivative carrying the *ftsA*_*Ng*_ gene fragment encoding amino acids 1–162	[33]
pcI_434_-AT2	pcI_434_ derivative carrying the *ftsA*_*Ng*_ gene fragment encoding amino acids 1–194	[33]
pcI_434_-AT3	pcI_434_ derivative carrying the *ftsA*_*Ng*_ gene fragment encoding amino acids 1–230	[33]
pcI_434_-AT4	pcI_434_ derivative carrying the *ftsA*_*Ng*_ gene fragment encoding amino acids 231–301	[33]
pcI_434_-AT5	pcI_434_ derivative carrying the *ftsA*_*Ng*_ gene fragment encoding amino acids 302–414	[33]
pcI_434_-AT6	pcI_434_ derivative carrying the *ftsA*_*Ng*_ gene fragment encoding amino acids 351–414	[33]
pET30a	Kan^R^ P_T7_::6Xhis	EMD Millipore, Billerica, MA
pETA	pET30a derivative carrying the *ftsA*_*Ng*_ gene	This study
pETQ	pET30a derivative carrying the *ftsQ*_*Ng*_ gene	This study
pETZ	pET30a derivative carrying the *ftsZ*_*Ng*_ gene	This study
pGEX2T	Amp^R^P_tac_::*gst*::*lacIq*	Amersham Bioscience, Uppsala, Sweden
pGEXA	pGEX2T derivative carrying the *ftsA*_*Ng*_ gene	This study
pGEXN	pGEX2T derivative carrying the *ftsA*_*Ng*_ gene	This study

The expression of *ftsA*_Ng_, *ftsZ*_Ng_ and *zipA*_Ng_ from B2H constructs was verified by Western blot analysis using appropriate antibodies prepared in our lab using previously described methods [33]. These proteins were expressed from the vectors under the conditions tested (data not shown). The expression of these proteins indicated that any negative B2H interactions involving them was not a function of lack of expression.

To ascertain what subdomains of FtsA_Ng_ interacted with gonococcal cell division proteins FtsZ_Ng_, FtsQ_Ng_, FtsW_Ng_, or FtsN_Ng_, six previously created truncations of the protein (T1, T2, T3, T4, T5, and T6; Additional file 1: Figure S1) were used [33]. Plasmid constructs for B2H assays were previously generated [33].

B2H assays were performed as described previously [24]. This assay is based on the reconstitution of a chimeric repressor that binds to the 434/P22 hybrid operator and represses the expression of a downstream *lacZ* gene in *E. coli* R721. Each gene tested for a potential interaction was cloned into pcI_p22_ and pcI_434_ and recombinant constructs were transformed into *E. coli* R721 either singly or in combination. *N. gonorrhoeae* FtsZ self-interaction was used as positive control. R721 without plasmids and single plasmid transformants were used as negative controls. R721 without plasmids had a β-galactosidase activity of 2504 ± 34 Miller units. The β-galactosidase activity of each combination was compared to that of R721. Values of less than 50% (<1250 Miller Units) indicate a positive interaction between two proteins, while values of more than 50% (>1250 Miller Units) indicate a negative interaction [24]. Statistical analyses were performed using the unpaired Student t-test. Standard deviations were determined for the mean value of Miller units where three independent experiments were performed.

### Construction and purification of his-fusion proteins

For His-fusion constructs, full-length *ftsA, ftsQ,* and *ftsZ* were PCR-amplified from *N. gonorrhoeae* CH811 genomic DNA using primer pairs P15/P16, P17/P18 and P19/P20 (Table 2), respectively. PCR amplicons were digested with EcoRI and BglII and ligated into pre-digested pET30a, to create pETA, pETQ, and pETZ. Plasmid pETA was transformed into *E. coli* C41 (DE3) and plasmids pETQ and pETZ were transformed into *E. coli* BL21 (DE3). The overexpression of all fusion proteins was induced with 400 μM IPTG, at 30 °C, for 2 h. Purification of His-FtsA_Ng_, His-FtsZ_Ng,_ and His-FtsQ_Ng_ was completed using His•Bind® Resin (EMD Millipore, Billerica, MA), following the manufacturer’s instructions. His-FtsZ_Ng_ was further treated with thrombin protease (EMD Millipore, Billerica, MA), overnight, at 4 °C, to cleave the N-terminal His tag. Thrombin was removed using 100 μl of p-aminobenzamidine-agarose (Sigma #A7155). FtsZ was dialyzed against MES buffer (50 mM MES, 300 mM KCl, 10 mM MgCl_2_, pH 7.5) prior to use in FtsZ polymerization experiments [34].

### GST pull-down assay

For GST fusion constructs, full-length *ftsA* and *ftsN* were PCR-amplified, from *N. gonorrhoeae* CH811, using primer pairs P21/P18 and P22/P23 (Table 2), respectively. The *ftsA* amplicon was digested with BamHI and EcoRI and ligated into pre-digested pGEX2T, to create pGEXA (Table 3). The *ftsN* amplicon was digested with EcoRI and XhoI and ligated into pre-digested pGEX2T, producing pGEXN (Table 3). Plasmids pGEXA and pGEXN were transformed into *E. coli* C41 (DE3) and *E. coli* BL21 (DE), respectively. Overexpression of GST-FtsA and GST-FtsN was accomplished by induction with either 400 μM or 800 μM of IPTG, respectively, at 30 °C, for 2 h. Purification of GST-FtsA and GST-FtsN was carried out using GST•Bind™ Resin (EMD Millipore, Billerica, MA), following the manufacturer’s instructions.

Purified GST-fusion and His-fusion proteins were incubated with pre-equilibrated GST•Bind™ Resin in phosphate buffered saline (PBS) buffer (137 mM NaCl, 2.7 mM KCl, 10 mM Na_2_HPO_4_, 1.8 mM KH_2_PO_4_, 0.5% Triton-X100, 1 mM DTT, pH 7.9) at 4 °C overnight. Pre-purified GST was used as a negative control. The pre-bound resin was collected by centrifugation and washed in PBS three times. Bound proteins were dissociated from resin by adding 5X Laemmli buffer, separated by electrophoresis on 10% sodium dodecyl sulfate polyacrylamide gels (SDS-PAGE), and identified by Western blot using polyclonal anti-GST or anti-6 × His antibodies (Thermo Scientific; Waltham, MA), sequentially.

For FtsA_Ng_-FtsZ_Ng_ interactions, the GST pull-down assay was performed in MES buffer (50 mM MES-NaOH, 50 mM KCl, 10 mM MgCl_2_, 0.5% Triton-X100, 1 mM ATP, 2 mM GTP, pH 7.5) [34]. To promote the polymerization of FtsZ_Ng_ necessary for this interaction, FtsZ_Ng_ was treated with 2 mM GTP and 1 mM ATP, as described previously [34], before mixing with GST-FtsA_Ng_ and GST•Bind™ Resin.

All GST pull-down assays were performed minimally in duplicate.

### FtsZ polymerization assays

FtsZ_Ng_ polymerization was measured by 90° angle light scattering using a Dynapro-MS800 instrument (Wyatt Technology Corporation) with a wavelength of 310 nm and a slit width of 0.5 mm. MES buffer is optimal for FtsZ polymerization which is required to observe an FtsA-FtsZ interaction [34, 35]. FtsZ_Ng_ (~6 μM) in MES buffer (50 mM MES-NaOH, 50 mM KCl, 10 mM MgCl_2_, pH 7.5) was injected into a 45 ul quartz cuvette and warmed to 30 °C, prior to the measurement. Data were collected, for 4 min, from unpolymerized FtsZ_Ng_ to establish a baseline. GTP was then added to a final concentration of 2 mM and data were collected every 5 s for 25 min. Data were recorded and analyzed using Dynamics v5 software.

Negative stain electron microscopy was used to visualize FtsZ_Ng_ polymers. 5 μl of FtsZ (6 μM) with, or without, GTP (final concentration 2 mM) was incubated, at 30 °C, for 5 min. The mixture was placed on a carbon-coated copper grid (400 mesh size) for 2 min and then blot dried. The grid containing FtsZ_Ng_ was stained with 1% uranyl acetate, blotted, and air-dried for 3 h. Polymers were visualized and photographed using a Hitachi transmission electron microscopy HT7700.

### Surface plasmon resonance (SPR)

Protein interactions were examined by SPR using a Bio-Rad XPR36 (Bio-Rad Laboratories) instrument and a ProteOn™ HTE Sensor Chip (Bio-Rad Laboratories). The chip surface was regenerated by injection of 0.5% SDS, 50 mM NaOH, 100 mM HCl and 300 mM EDTA, at a flow rate of 30 μl/min, for 120 s. Activation was performed using 500 μM of NiSO_4_.

For FtsA_Ng_-FtsN_Ng_ and FtsA_Ng_-FtsQ_Ng_ SPR experiments, ligands (i.e. His-FtsN_Ng_ for FtsA_Ng_-FtsN_Ng_, and His-FtsQ_Ng_ for FtsA_Ng_-FtsQ_Ng_ interactions) were immobilized onto the sensor chip at a concentration of 200 nM. A two-fold dilution series of the analyte (FtsA_Ng_), in PBS buffer with Tween-20 (PBST; 137 mM NaCl, 2.7 mM KCl, 10 mM Na_2_HPO_4_, 1.8 mM KH_2_PO_4_, 0.1% BSA, 0.05% Tween-20, pH 7.9), was injected at a flow rate of 30 μl/min over the surface of the chip for 120 s. This was followed by an injection of PBST buffer for 300 s. Negative controls comprised a reference channel flowed with PBST buffer, and a chip surface immobilized with either FtsQ_Ng_ or FtsN_Ng_ flowed with GST in PBST.

For the FtsA_Ng_-FtsZ_Ng_ interaction, the SPR binding assay was performed using MES buffer, supplemented with 0.1% BSA, 0.05% Tween-20, and 1 mM ATP were added with the pH adjusted to 7.5. FtsA_Ng_ was immobilized on the chip surface as described above. Each 120-s injection of polymerized FtsZ_Ng_ was followed by an injection of supplemented MES buffer for 300 s for dissociation. Negative controls included a reference channel which was flowed with MES buffer containing 2 mM GTP, and the FtsA_Ng_-immobilized chip surface flowed with GST in supplemented MES instead of polymerized FtsZ_Ng_.

All SPR data was analyzed using ProteOn Manager™ (Bio-Rad Laboratories). The sensorgram (i.e. a graph of the response unit versus time) was first subtracted by the response units (RU) of the reference channel, with no immobilized ligands, to reduce the non-specific binding signals between analyte and empty chip surface. Then, the sensorgram was subtracted with the RU signal with running buffer and ligand immobilized on the chip. Association and disassociation constants were obtained using the Langmuir 1:1 kinetic fit model, by nonlinear regression, using ProteOn Manager™. Each protein pair was tested minimally in duplicate.

## Results

### Identification of *N. gonorrhoeae* cell division protein interactions by bacterial two-hybrid assay

Using B2H assays, we investigated 28 potential interactions among eight gonococcal divisome proteins including FtsZ, FtsA, ZipA, FtsK, FtsQ, FtsI, FtsW, and FtsN. The results (Table 4) show that nine interactions, FtsZ-FtsA, FtsZ-FtsK, FtsZ-FtsW, FtsA-FtsK, FtsA-FtsQ, FtsA-FtsW, FtsA-FtsN, FtsI-FtsW, and FtsK-FtsN, displayed a residual β-galactosidase activity lower than 50%, indicating a positive interaction between these proteins in *N. gonorrhoeae*. The interaction between FtsA_Ng_ and FtsN_Ng_ had the lowest residual β-galactosidase activity (24%), indicating the strongest interaction. This was followed by FtsA_Ng_-FtsK_Ng_ (30%), FtsN_Ng_-FtsK_Ng_ (31%), FtsI_Ng_-FtsW_Ng_ (35%), FtsZ_Ng_-FtsW_Ng_ (39%), FtsA_Ng_-FtsZ_Ng_ (40%), FtsZ_Ng_-FtsK_Ng_ (41%), FtsA_Ng_-FtsW_Ng_ (45%), and FtsA_Ng_-FtsQ_Ng_ (48%) interactions. ZipA_Ng_ did not directly interact with other cell division proteins as the residual β-galactosidase activity of all interactions was above 50% (Table 4).

**Table 4:**
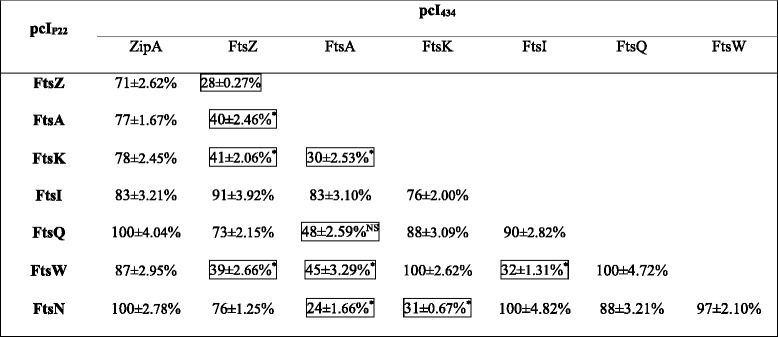
Interactions between eight cell division proteins in *N. gonorrhoeae* as determined by B2H assay

By comparison to positive controls (*E. coli* R721 without plasmids), interactions with less than 50% of residual ß-galactosidase activity (framed) were considered as positive. FtsZ_Ng_ self-interaction was used as a positive control. The numbers represent percentage of mean ß-galactosidase activity, ± standard deviation

^*^Statistically significant (*P* ≤ 0.05); NS: not statistically significant (*P* > 0.05)

### GST pull-down of FtsA_Ng_-FtsQ_Ng_, FtsA_Ng_-FtsZ_Ng_ and FtsA_Ng_-FtsN_Ng_ interactions

**Fig. 1:**
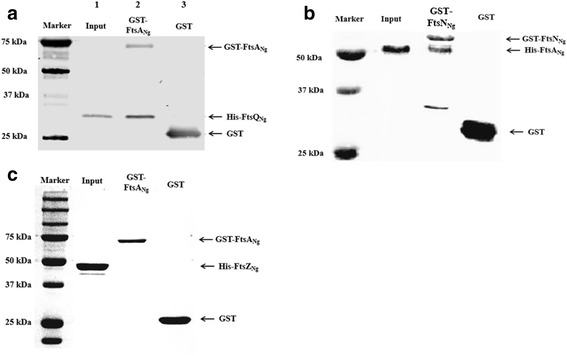
Interactions of FtsA_Ng_ with FtsQ_Ng_, FtsN_Ng_ and FtsZ_Ng_ by GST pull-down. **a** GST pull down between His-FtsQ_Ng_ and GST-FtsA_Ng_. Lane 1: His-FtsQ_Ng_ input; Lane 2: GST-FtsA_Ng_ and His-FtsQ_Ng_ mixture; Lane 3: GST and His-FtsQ_Ng_ mixture; **b** GST pull down between His-FtsA_Ng_ and GST-FtsN_Ng_. Lane 1: His-FtsA_Ng_ input; Lane 2: GST-FtsN_Ng_ and His-FtsA_Ng_ mixture, GST-FtsN_Ng_ was loaded with GST and GST-FtsN_Ng_ degradation products; Lane 3: GST and His-FtsA_Ng_ mixture; **c** GST pull down between His-FtsZ_Ng_ and GST-FtsA_Ng_. Lane 1: His-FtsZ_Ng_ input; Lane 2: GST-FtsA_Ng_ and His-FtsZ_Ng_ mixture; Lane 3: GST and His-FtsZ_Ng_ mixture; His-tagged fusion proteins were visualized using anti-6 × His antibody; GST and GST-tagged fusion proteins were visualized using anti-GST antibody

To confirm the results of selected B2H assays, we examined several interactions (i.e. FtsQ_Ng_-FtsA_Ng_, FtsA_Ng_-FtsN_Ng_, FtsA_Ng_-FtsZ_Ng_) using GST pull-down assays. GST pull-down results (Fig. 1a) showed that His-FtsQ_Ng_ was pulled down by GST-FtsA_Ng_, but not GST itself (negative control), indicating an interaction between FtsA_Ng_ and FtsQ_Ng_. Using similar evaluation criteria, we ascertained that His-FtsA_Ng_ was pulled down by GST-FtsN_Ng_, indicating an interaction between these two proteins (Fig. 1b).

The interactions of FtsA_Ng_ and FtsZ_Ng_ from *E. coli* in vitro requires the presence of both ATP and GTP [35]. GTP promotes FtsZ polymerization, and ATP is necessary for FtsA to interact with FtsZ, but not for FtsZ polymerization [36, 37]. The presence of FtsZ_Ng_ polymers in MES buffer was determined by transmission electron microscopy (TEM) and dynamic light scattering (DLS; Additional file 2: Figure S2). GST pull-down assay did not detect an interaction between FtsA_Ng_ and FtsZ_Ng_ in the presence of 1 mM ATP and 2 mM GTP (Fig. 1c). This result was unexpected, given our B2H results and the commonality of FtsA-FtsZ interaction in other bacterial species [24, 25, 38, 39], as ascertained by different in vivo assays (i.e. B2H, yeast two-hybrid, chemical cross-linking with co-immunoprecipitation).

### Surface plasmon resonance evaluation of FtsA_Ng_-FtsQ_Ng_, FtsA_Ng_-FtsZ_Ng_ and FtsA_Ng_-FtsN_Ng_ interactions

**Fig. 2:**
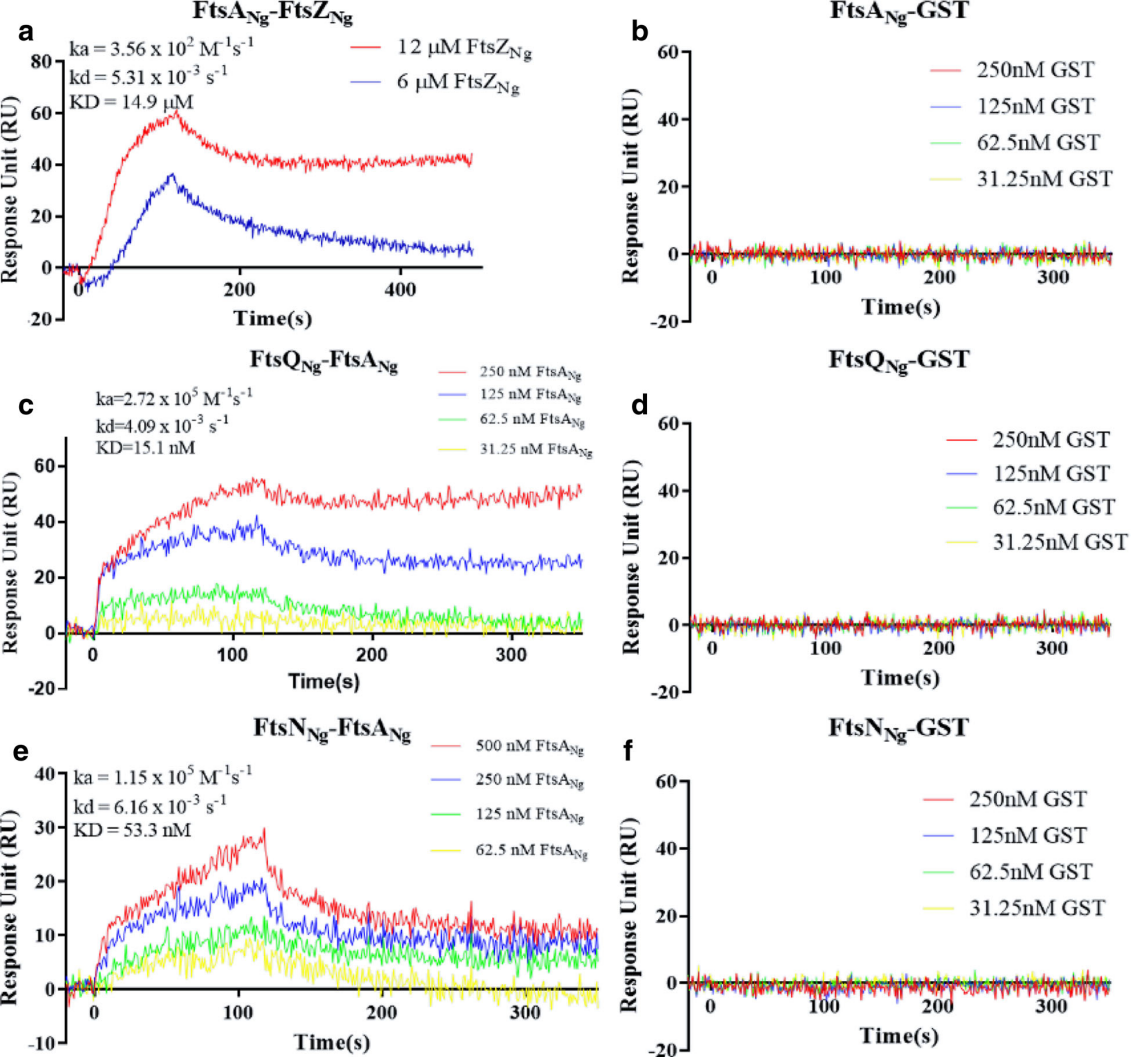
SPR measurement for *N. gonorrhoeae* FtsA-FtsZ, FtsQ-FtsA and FtsA-FtsN interactions. **a** 6 and 12 μM of FtsZ_Ng_ were analyzed for interaction with FtsA_Ng_; **b** Negative interaction between FtsA_Ng_ and GST; **c** FtsA_Ng_ at different concentrations (31.25, 62.5, 125 and 250 nM) were measured for binding affinity to FtsQ_Ng_; **d** Negative interaction between FtsQ_Ng_ and GST; **e** FtsN_Ng_ at different concentrations (62.5, 125, 250 and 500 nM) was analyzed for interaction with FtsA_Ng_; **f** Negative interaction between FtsN_Ng_ and GST. Association and disassociation constants were obtained using the Langmuir 1:1 kinetic fit model by nonlinear regression using ProteOn Manager™ (Bio-Rad Laboratories)

Surface plasmon resonance (SPR) was used to confirm selected gonococcal cell division protein-protein interactions in real-time. SPR was used to evaluate the interactions of FtsA_Ng_ with FtsZ_Ng_ because of the conflicting results observed with B2H and GST pull-down assays. GTP was added to promote FtsZ_Ng_ polymerization (Additional file 2: Figure S2). The sensorgram indicated that FtsZ_Ng_ interacted with FtsA_Ng_ at concentrations of 6 μM and 12 μM (Fig. 2a), but not at concentrations lower than 6 μM (data not shown). Kinetic analysis showed that the FtsA_Ng_-FtsZ_Ng_ interaction had a slow association (ka = 3.56 × 10^2^ M^−1^ s^−1^) and a significant disassociation activity (kd = 5.31 × 10^−3^ s^−1^), giving a KD value of 14.9 μM. This suggested that the interaction between FtsA_Ng_ and FtsZ_Ng_ was likely transient. When GTP was absent from the FtsZ_Ng_ protein solution, no binding was detected between FtsA_Ng_ and FtsZ_Ng_ (data not shown). The sensorgram of the interaction between FtsA_Ng_ and the negative control (GST) also showed no binding activity (Fig. 2b), indicating the specificity of the SPR results for the interaction of FtsA_Ng_ with FtsZ_Ng_.

For the SPR analysis of the FtsA_Ng_-FtsQ_Ng_ interaction, FtsA_Ng_ was tested using various concentrations (from 31.25 nM to 250 nM; Fig. 2c). At 0 s, the association of FtsA_Ng_ and FtsQ_Ng_ was observed immediately following injection of the FtsA_Ng_ solution onto the FtsQ_Ng_-labeled chip surface, with a rapid increase of response units (ka = 2.72 × 10^5^ M^−1^ s^−1^; Fig. 2c). This indicated a fast binding event between the two proteins. Disassociation between FtsA_Ng_ and FtsQ_Ng_ was not significant (kd = 4.09 × 10^−^3 s^−1^), suggesting this interaction was strong and stable (KD = 15.1 nM). The negative control, using non-interacting GST, did not cause any change in the response units (Fig. 2d).

The FtsA_Ng_-FtsN_Ng_ interaction was observed with an increasing concentration of FtsA_Ng_ (62.5 nM, 125 nM, 250 nM and 500 nM; Fig. 2e). His-FtsN_Ng_ had a binding affinity (KD) of 53.3 nM with FtsA_Ng_. The association and disassociation constants were 1.15 × 10^5^ M^−1^ s^−1^, and 6.16 × 10^−3^ s^−1^, respectively (Fig. 2e), indicating a strong interaction between FtsA_Ng_ and FtsN_Ng_. The injection of non-interacting GST onto the FtsN_Ng_ immobilized chip surface did not cause any change in the response units (Fig. 2f).

### The 2A and 2B subdomains of FtsA_Ng_ interacts with FtsZ_Ng_, FtsN_Ng_, FtsW_Ng_ and FtsQ_Ng_

**Fig. 3:**
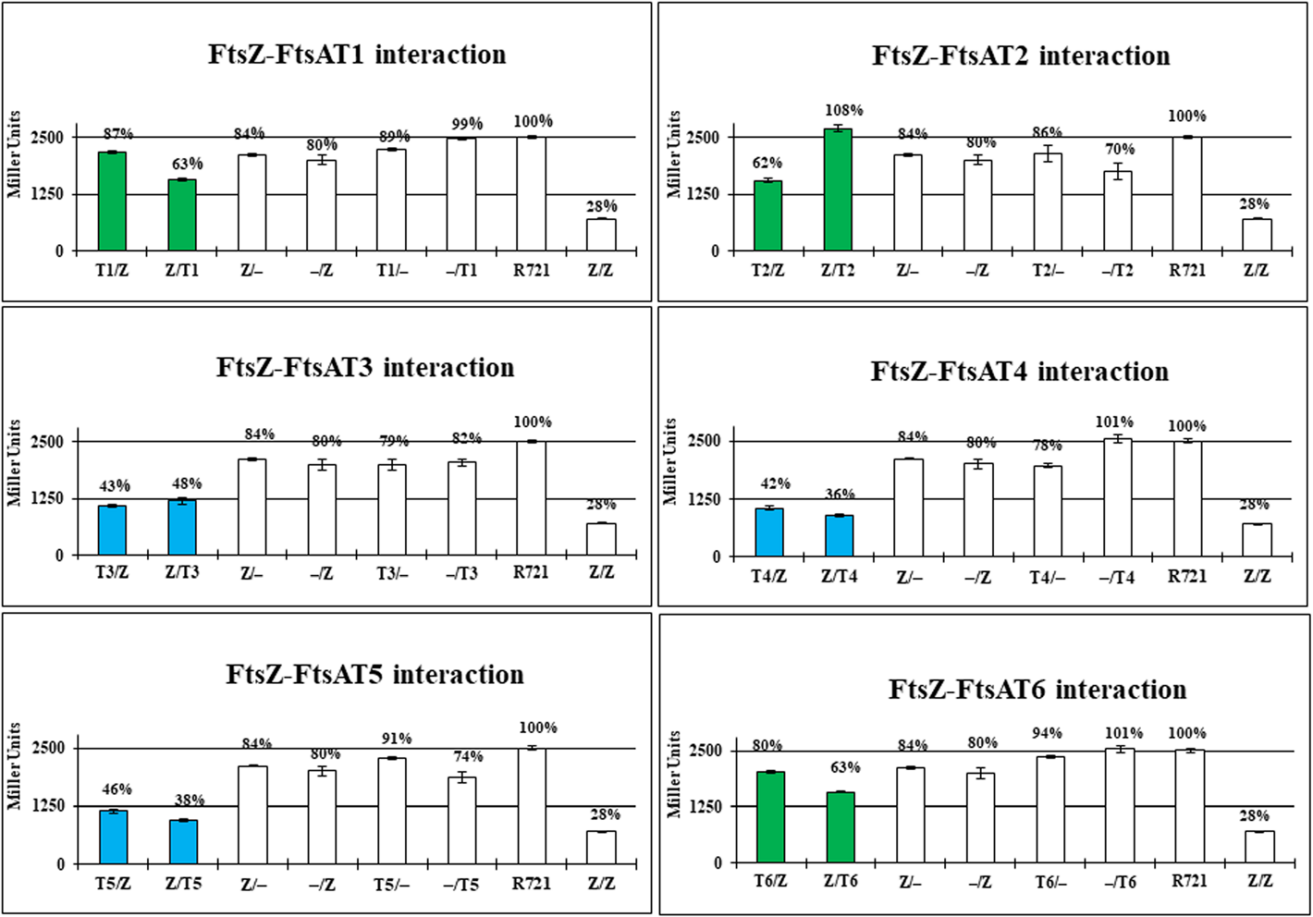
Interactions between FtsA_Ng_ truncations (T1, T2, T3, T4, T5 and T6) and FtsZ_Ng_ (Z) by B2H assays. R721 without plasmids and single transformants were used as negative controls. R721 without plasmids had a β-galactosidase activity of 2504 ± 34 Miller units. FtsZ_Ng_ self-interaction was used as a positive control. Values of less than 50% (<1250 Miller Unites) indicate a positive interaction between two proteins (blue bars) while values of more than 50% (>1250 Miller Unites) indicate a negative interaction (green bars) positive and negative controls are labeled in white (white bar)

**Fig. 4:**
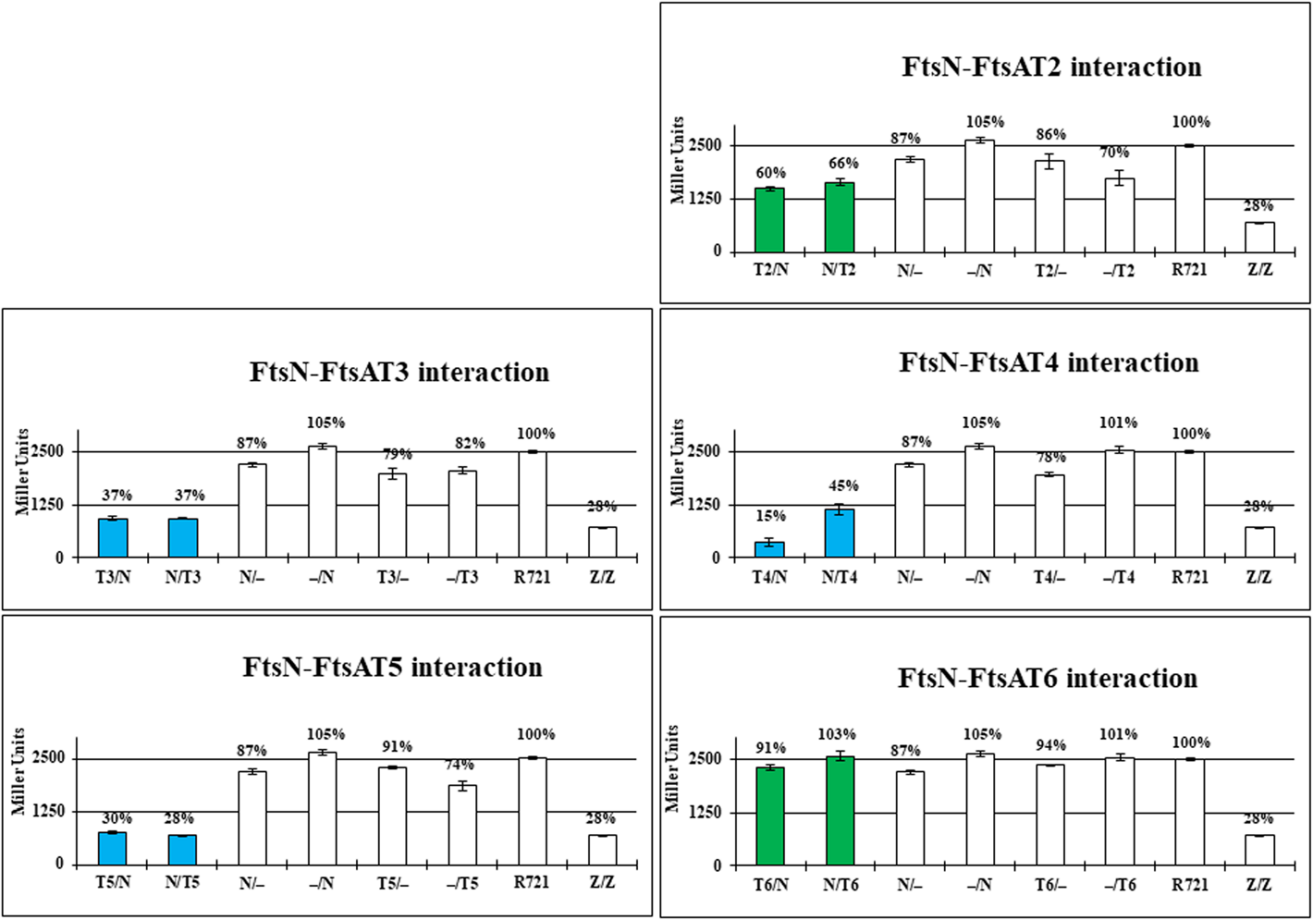
Interactions between FtsA_Ng_ truncations (T2, T3, T4, T5 and T6) and FtsN_Ng_ (N) by B2H assays. Values of less than 50% (<1250 Miller Unites) indicate a positive interaction (blue bars) while values of more than 50% (>1250 Miller Unites) indicate a negative interaction (green bars)

**Fig. 5:**
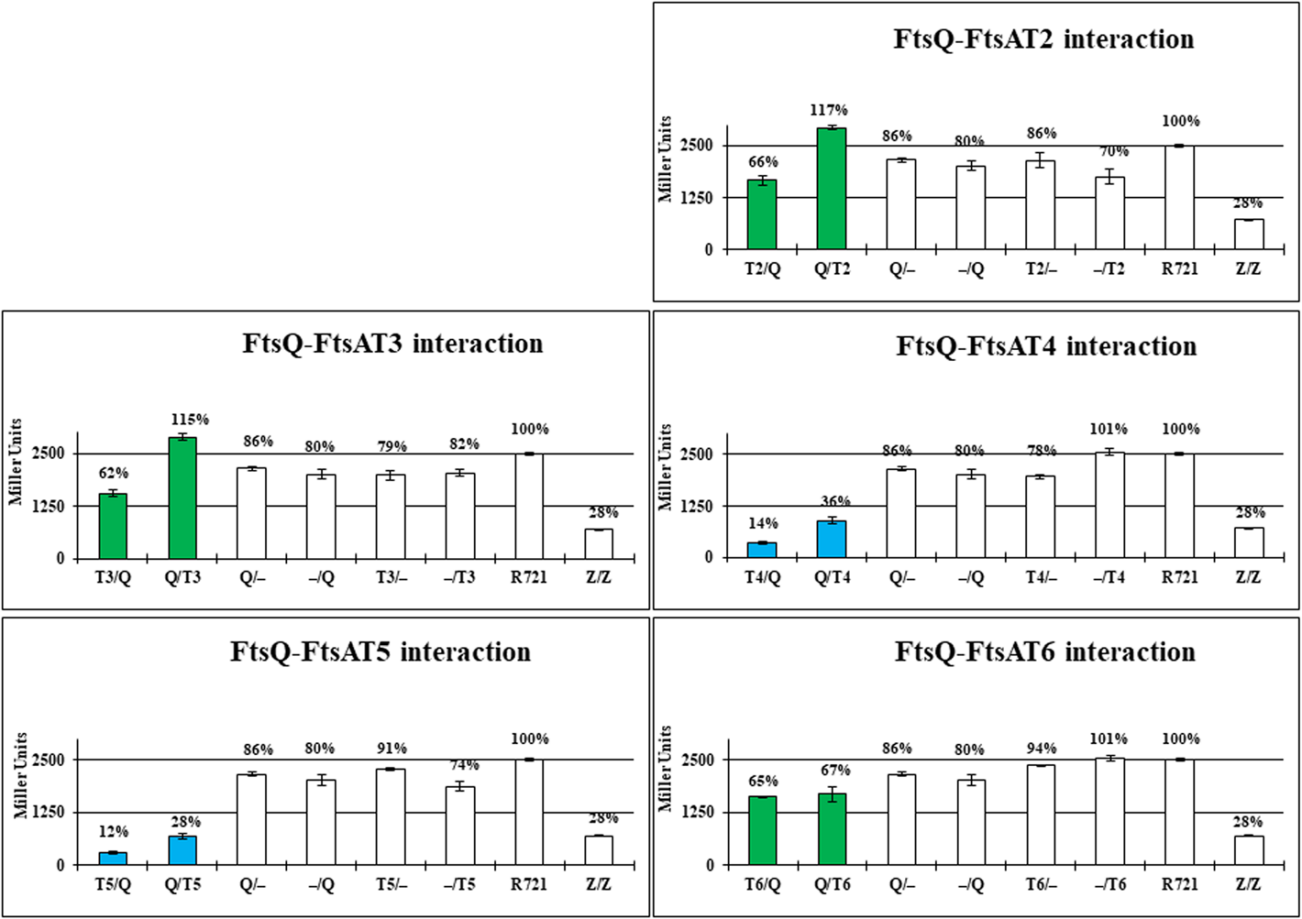
Interactions between FtsA_Ng_ truncations (T2, T3, T4, T5 and T6) and FtsQ_Ng_ (Q) by B2H assays. Values of less than 50% (<1250 Miller Unites) indicate a positive interaction between two proteins (blue bars) while values of more than 50% (>1250 Miller Unites) indicate a negative interaction between the two proteins (green bars)

Since FtsA_Ng_ interacted with FtsZ_Ng_, FtsQ_Ng_, FtsW_Ng_, and FtsN_Ng_, we further examined the interaction regions of FtsA_Ng_ with these four proteins using B2H assays. Based on FtsA_Ng_ homology modeling, six FtsA_Ng_ truncations (T1-T6) were created (Additional file 1: Figure S1), which contained one or more FtsA_Ng_ subdomains [33]. FtsZ_Ng_ self-interaction was used as a positive control. And negative controls included *E. coli* R721 without plasmids or carrying each single recombinant B2H vector in which the gene of interest had been cloned. FtsA_Ng_ truncations T3, T4, and T5 interacted with FtsZ_Ng_ and FtsN_Ng_ (Figs. 3 and 4, blue bars). FtsA_Ng_ truncations T1, T2, and T6 did not show an interaction with these proteins (Figs. 3 and 4, green bars). The T4 and T5 truncations included the 2B and 2A_2_ subdomains of FtsA_Ng_, suggesting that these subdomains of FtsA_Ng_ interacted with both FtsZ_Ng_ and FtsN_Ng_. The T3 construct contained also contained the 2A_1_ subdomain of FtsA_Ng_, as compared to truncations T1 and T2, indicating that this subdomain was also involved in interactions with FtsZ_Ng_ and FtsN_Ng_. FtsQ_Ng_ interacted only with the T4 and T5 truncations of FtsA_Ng_ (Fig. 5, blue bars), indicating that the 2B and 2A_2_ subdomains, but not the 2A_1_ subdomain, were required for the FtsA_Ng_-FtsQ_Ng_ interaction. Only the T5 truncation of FtsA_Ng_ interacted with FtsW_Ng_, suggesting that 2A_2_ subdomain was involved in the interaction with FtsW_Ng_ (Additional file 3: Figure S3). In summary, these results showed that the 2A_1_, 2A_2_ and 2B subdomains of FtsA_Ng_ are required for its interaction with FtsN_Ng_ and FtsZ_Ng_. The FtsA_Ng_ 2A_2_ and 2B subdomains are required for interaction with FtsQ_Ng_, and the 2A_2_ subdomain is involved in the interaction with FtsW_Ng_.

## Discussion

**Fig. 6:**
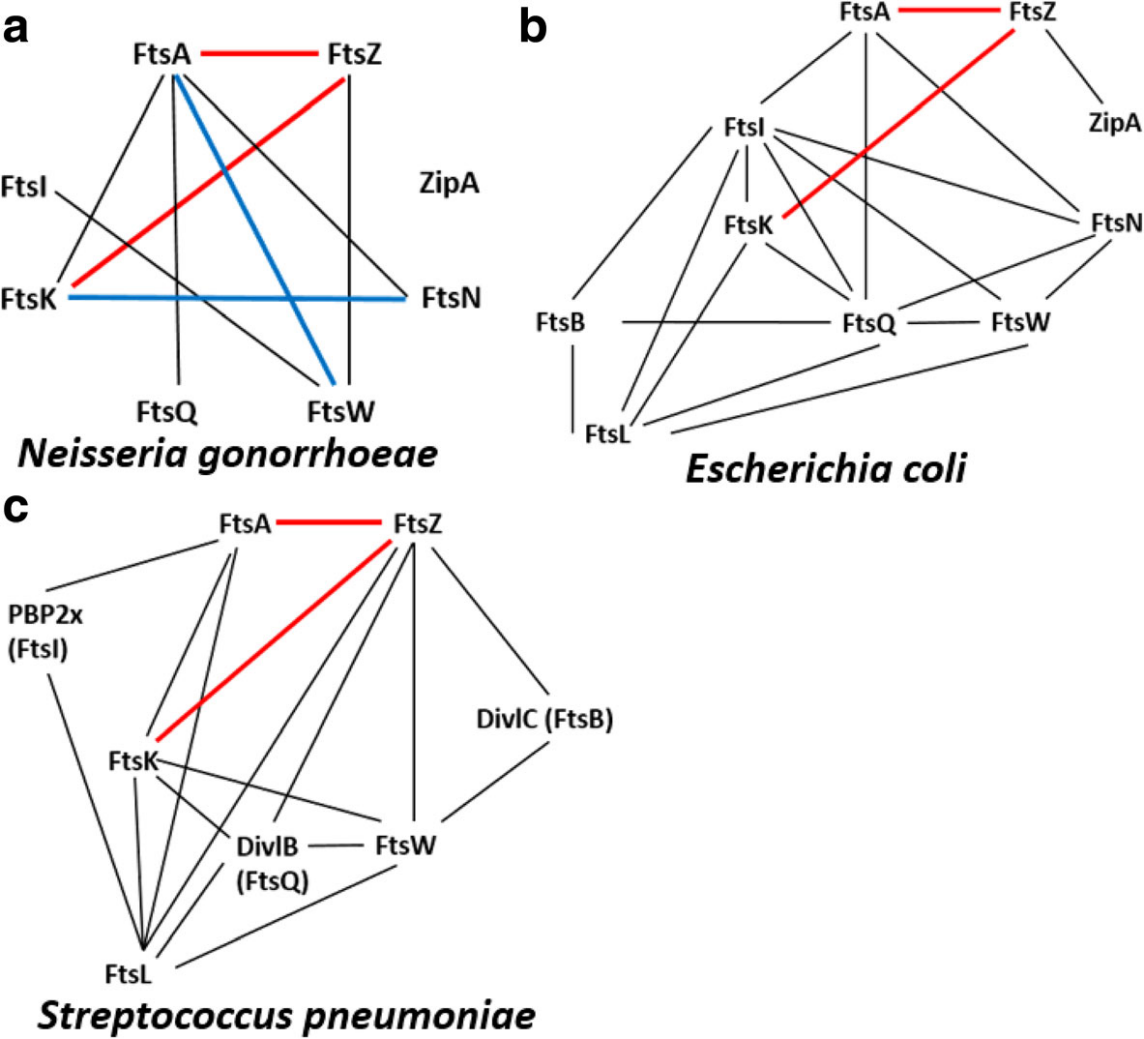
Cell division interactomes of **a** *N. gonorrhoeae*, **b** *E. coli* [23, 24], and **c** *S. pneumoniae* [25]. Red lines indicate common interactions; blue lines indicate unique interactions in *N. gonorrhoeae*

The *N. gonorrhoeae* cell division interactome described in our study is the third cell division interaction network identified in bacteria, in addition to *E. coli* and *S. pneumoniae* (Fig. 6a) [23–25]. Compared to the other two interactomes (Fig. 6b and c), fewer interaction protein pairs are identified in *N. gonorrhoeae* (Fig. 6a). Only nine interactions are present among the eight divisome proteins tested in *N. gonorrhoeae*, while *E. coli* and *S. pneumoniae* have 21 and 17 interactions among ten and eight divisome proteins, respectively [24, 25].

The development of all three cell division interactomes was based on interaction data obtained from the same B2H system [24, 25] The *E. coli* interactome was developed using B2H results exclusively while the *S. pneumoniae* study also applied co-immunoprecipitation to verify selected B2H positive interaction pairs [24, 25]. In our study, we used a combination of GST pull-down and surface plasmon resonance to further study selected positive B2H interactions.

Two interactions, FtsA-FtsZ and FtsZ-FtsK, are conserved in the cell division interactomes of *N. gonorrhoeae*, *E. coli* and *S. pneumoniae* (Fig. 6, red lines). The FtsA-FtsZ interaction is a common interaction in prokaryotes [24, 25, 39–41]. Both our B2H and SPR results confirmed this interaction in *N. gonorrhoeae*. A proper ratio between FtsA and FtsZ is crucial for the interaction in *E. coli* [42] and our SPR results support this finding; FtsA_Ng_ interacts with FtsZ_Ng_ only when its concentration is higher than 6 μM (Fig. 3b), indicating that the interaction requires a critical concentration threshold. Our SPR results further showed that interaction between FtsA_Ng_ and FtsZ_Ng_ was transient, a result warranting further study to fully understand its implications for divisome formation in *N. gonorrhoeae*. Unexpectedly, the GST pull-down assay, an in vitro assay, did not detect an FtsA_Ng_-FtsZ_Ng_ interaction. We believe that this “false negative” in vitro result was caused by the requirement of a membrane/solid surface support for the interaction to anchor FtsA [35, 43, 44].

The interaction of FtsZ with FtsK has been observed in *N. gonorrhoeae*, *E. coli*, *S. pneumoniae, B. subtilis* and *C. crescentus* [24, 25, 45, 46]. The C-terminus of FtsK is required for proper DNA segregation in *E. coli* [47]. The absence of an FtsZ-FtsK interaction in both *E. coli* and *C. crescentus* caused abnormal chromosome segregation and cell filamentation [45, 48]. This suggests that the FtsZ-FtsK interaction connects the cell division process with chromosome segregation, by ensuring that the replicated chromosome is cleared from the division site.

The FtsA-FtsW interaction has been observed only in *N. gonorrhoeae* (Fig. 6, blue lines). Since FtsW is a membrane protein and difficult to purify, we did not verify the interaction by GST pull-down and SPR assays. However, we performed additional B2H assays to identify which subdomains of FtsA were involved in its interaction with FtsW (Additional file 3: Figure S3) and showed that the 2A_2_ subdomain of FtsA strongly interacts with FtsW (Additional file 3: Figure S3). FtsW, an inner membrane protein, is required in *E. coli* for the recruitment of FtsI and the translocation of the cell well precursor, lipid II [20, 21, 49, 50]. An FtsI-FtsW protein interaction has been observed in *E. coli*, *Streptomyces coelicolor*, and *Mycobacterium tuberculosis* [21, 51, 52]. Interestingly, we discovered that FtsI_Ng_ only interacts with FtsW_Ng_, suggesting that its localization may depend on this protein.

The importance of the unique FtsK_Ng_-FtsN_Ng_ interaction in *N. gonorrhoeae*, as determined by B2H, is not clear (Fig. 6, blue lines). In *E. coli*, FtsN is the last protein, of ten essential cell division proteins, recruited to the division site to initiate cell constriction [53, 54]. A previous study suggested that *E. coli* FtsN and FtsK stabilize the Z-ring cooperatively, without direct interactions [55]. Since the FtsK-FtsN interaction is present in *N. gonorrhoeae*, their joint involvement in gonococcal cell division requires further investigation.

ZipA_Ng_ did not interact with any other gonococcal cell division protein. In *E. coli*, ZipA only interacts with FtsZ, and is required for downstream protein recruitment, including FtsK, FtsQ, FtsL, and FtsN [24, 56]. One report suggested that ZipA_Ng_ is a homologue of the *E. coli* protein with high similarity in its key domains [57]. Although ZipA_Ng_ complemented a conditional *zipA* mutant in *E. coli*, it did not fully restore a wild type phenotype in this strain [57]. Given these data, the role of ZipA in gonococcal cell division remains to be elucidated.

In *N. gonorrhoeae*, the existence of FtsL_Ng_ is unclear due to its low homology with *E. coli* FtsL [58]. An open reading frame (ORF) located between *mraW* and *ftsI* in the *dcw* cluster of *N. gonorrhoeae* was reported by Francis et al. [7] and they reported that it was not a coding ORF. Snyder et al. [58] named the same ORF *ftsL*. Because this ORF shares only 17% amino acid similarity to its *E. coli* homologue, we considered that it was not a functional ORF and did not test its interaction with other gonococcal cell division proteins.

*N. gonorrhoeae* lacks FtsB [7]; thus, the protein complex FtsQ-B-L, present in other species, such as *E. coli*, *S. pneumoniae* and *B. subtilis*, would not be formed in *N. gonorrhoeae* [59–61]. This protein complex has been described as a bridge connecting FtsK and the FtsI-FtsW complex in *E. coli* [18]. A recent study suggests that the *E. coli* FtsQ-B-L complex acts as a signal transmitter for cell wall remodeling and constriction, which is mediated by direct interactions with the FtsI-W complex and FtsN [19]. In *S. pneumoniae*, the FtsQ homologue, DivIB, interacts with FtsK_Sp_, FtsL_Sp,_ and FtsW_Sp_ [25]. Interestingly, our B2H data show that FtsQ_Ng_ only interacts with FtsA_Ng_, suggesting that the function of FtsQ_Ng_ in cell division in *N. gonorrhoeae* may be distinct.

There are several models for bacterial cell constriction. One *E. coli* model suggests that the force that drives constriction comes from septal peptidoglycan synthesis [62]. In this model, the FtsA_Ec_-FtsN_Ec_ interaction activates peptidoglycan synthesis by direct or indirect interaction with FtsI_Ec_ [63]. Another *E. coli* model suggests that the energy generated from FtsZ-mediated GTP hydrolysis drives cell constriction [43]. We observed an FtsA_Ng_-FtsN_Ng_ interaction in *N. gonorrhoeae*. However, there is no further evidence supporting either model of cell constriction in *N. gonorrhoeae* at this time.

The non-essential proteins, FtsE_Ng_ and FtsX_Ng_, are also implicated in cell division in *N. gonorrhoeae* [64]. Similarly, in *E. coli*, FtsE and FtsX are non-essential for cell division under conditions of high osmotic pressure [65]. Gonococcal FtsE and FtsX have high similarity in amino acid sequence to known homologues in other species [64]. In *E. coli*, the interaction between FtsE and FtsZ has a regulatory effect on the Z-ring [65]. Future research could focus on revealing the effects of FtsE_Ng_ and FtsX_Ng_ on cell division in *N. gonorrhoeae*.

The major issue interpreting B2H assay results is the empirical cut-off of 50% residual ß-galactosidase activity used to discriminate positive and negative interactions. In particular, values close to the cut-off could be interpreted as either false positive or negative results. To validate our B2H results, we used other B2H interactions to test which subdomains of FtsA_Ng_ interacted with FtsZ_Ng_, FtsN_Ng_, and FtsQ_Ng_. We determined that the 2A and 2B subdomains of FtsA_Ng_ interacted with FtsZ_Ng_, FtsQ_Ng_, and FtsN_Ng_. We also evaluated some positive interactions obtained by B2H using SPR and GST pull-down assays. The SPR method detects and measures weak or transient interactions, in real-time, with high sensitivity [66]. The SPR method showed a transient FtsA_Ng_-FtsZ_Ng_ interaction. GST pull-down assays, on the other hand, are ideal in detecting strong protein-protein interactions, as weak interactions may dissociate during the assay [67]. We consider this to be a reasonable explanation for our failure to confirm when the interaction of FtsA_Ng_ with FtsZ_Ng_ when using a GST pull-down assay.

To date, most of studies on cell division have been focused on model organisms (i.e. the Gram-negative rod *E. coli* and the Gram-positive rod *B. subtilis*) due to the abundant availability of tools for genetic manipulation [62]. Research on cell division in non-model organisms is expanding, and this includes studies with *N. gonorrhoeae* [7, 27]. For example, *Chlamydia trachomatis*, which lacks FtsZ, requires an actin-like protein, MreB, for cell division [68]. A gene cluster encoding three cell division proteins, named MldA, MldB, and MldC, was identified only in *Clostridium difficile* and its closely related bacteria [69]. Results from studies using non-model organisms suggest that cell division mechanisms are complex and vary in different organisms, reflecting vast biological diversity.

## Conclusions

In our research, we discovered that nine interactions among eight cell division proteins defined the cell division interactome of *N. gonorrhoeae*. In comparison with the published cell division interactomes of *E. coli* and *S. pneumoniae*, FtsA-FtsZ and FtsZ-FtsK interactions were common to all three bacteria. FtsK-FtsN and FtsA-FtsW interactions were only present in *N. gonorrhoeae*, suggesting that they play different roles in the cell division of this microorganism. ZipA_Ng_ did not interact with any other cell division proteins tested in this study, indicating that its role may differ as compared to its *E. coli* homologue. We also determined that the subdomains of FtsA_Ng_ which interacted with FtsQ_Ng_, FtsZ_Ng_, FtsW_Ng_, or FtsN_Ng_, differed from its *E. coli* homologue. This suggests that *N. gonorrhoeae* possesses a distinctive cell division interactome, and likely a different mechanism of cell division as compared to *E. coli* and other organisms.

## Additional files

Additional file 1: Figure S1. Schematic representation of *N. gonorrhoeae ftsA* and its truncations [33]. T1 (162aa, Met1-Ala162) contained the N-terminal 1A and 1C domains of *ftsA*_*Ng*_. T2 (194aa, Met1-Val194) included the N-terminal 1A, 1C and 1A domains of *ftsA*_*Ng*_. T3 (230aa, Met1-Ile230) included the N-terminal 1A, 1C, 1A and 2A_1_ domains of *ftsA*_*Ng*_. T4 (71aa, Pro231-Glu301) contained the 2B domain of *ftsA*_*Ng*_. T5 (114aa, Ile301-Leu414) contained the 2A_2_ and 1A C-terminal domains of *ftsA*_*Ng*_. T6 (64aa, Ala351-Leu414) contained the 1A C-terminal domain of *ftsA*_*Ng*_. (DOCX 30 kb)

Additional file 2: Figure S2. FtsZ_Ng_ polymerization assays. FtsZ_Ng_ polymers visualized by transmission electron microscope with(A) or without (B) 2 mM GTP in MES buffer (50 mM MES-NaOH, 50 mM KCl, 10 mM MgCl_2_, pH 7.5) at 30 °C. Solid arrows indicate FtsZ_Ng_ polymers. Scale bar indicates 100 nm. (C) Light scattering of FtsZ_Ng_ polymerization (6 μM) in MES buffer. (DOCX 211 kb)

Additional file 3: Figure S3. Interactions between FtsA_Ng_ truncations (T2, T3, T4, T5 and T6) and FtsW_Ng_ (W) by B2H assay. Values of less than 50% (<1250 Miller Units) indicate a positive interaction between two proteins (blue bars) while values of more than 50% (>1250 Miller Units) indicate a negative interaction (green bars). (DOCX 66 kb)

## Abbreviations

B2H: Bacterial two-hybrid
Bs: *Bacillus subtilis*
Cs: *Caulobacter crescentus*
*dcw*: division and cell wall
DLS: Dynamic light scattering
Ec: *Escherichia coli*
Ng: *Neisseria gonorrhoeae*
ORF: Open reading frame
Sp: *Streptococcus pneumoniae*
SPR: Surface plasmon resonance
TEM: Transmission electron microscopy
Y2H: Yeast two-hybrid
