# Supplementary figures and images for: The distinctive cell division interactome of Neisseria gonorrhoeae
Source: BMC Microbiol. 2017 Dec 12;17:232. doi: 10.1186/s12866-017-1140-1 (PMC5727935; doi:10.1186/s12866-017-1140-1)

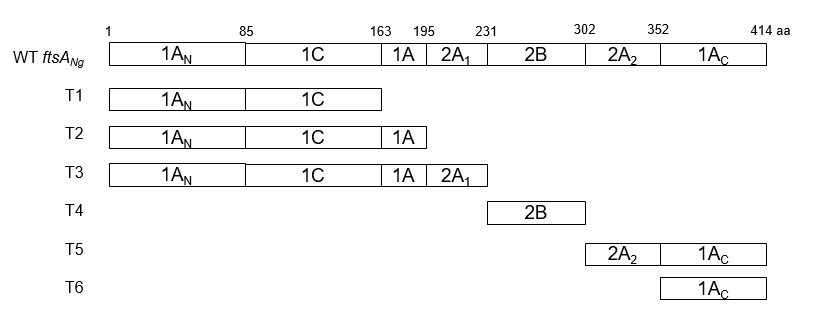
**Fig S1**

Supplement: Supplementary file 1 — Schematic representation of N. gonorrhoeae ftsA and its truncations [33]. T1 (162aa, Met1-Ala162) contained the N-terminal 1A and 1C domains of ftsA Ng. T2 (194aa, Met1-Val194) included the N-terminal 1A, 1C and 1A domains of ftsA Ng. T3 (230aa, Met1-Ile230) included the N-terminal 1A, 1C, 1A and 2A1 domains of ftsA Ng. T4 (71aa, Pro231-Glu301) contained the 2B domain of ftsA Ng. T5 (114aa, Ile301-Leu414) contained the 2A2 and 1A C-terminal domains of ftsA Ng. T6 (64aa, Ala351-Leu414) contained the 1A C-terminal domain of ftsA Ng. (DOCX 30 kb) [file 12866_2017_1140_MOESM1_ESM.docx]

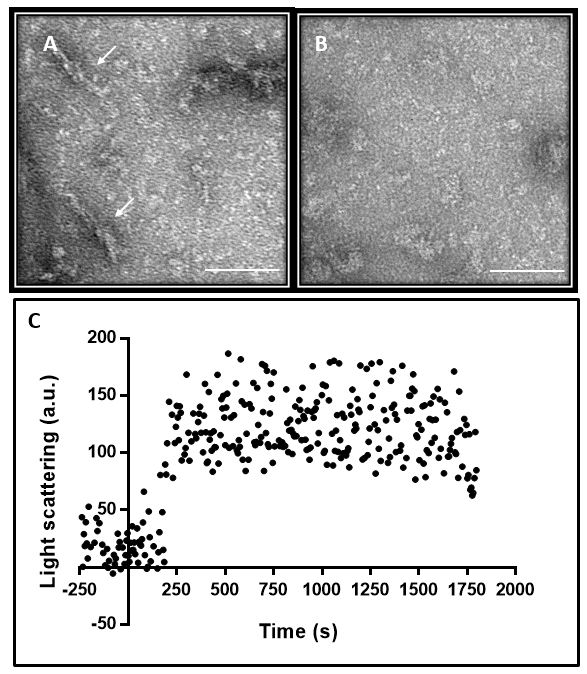
**Fig S2**

Supplement: Supplementary file 2 — FtsZNg polymerization assays. FtsZNg polymers visualized by transmission electron microscope with(A) or without (B) 2 mM GTP in MES buffer (50 mM MES-NaOH, 50 mM KCl, 10 mM MgCl2, pH 7.5) at 30 °C. Solid arrows indicate FtsZNg polymers. Scale bar indicates 100 nm. (C) Light scattering of FtsZNg polymerization (6 μM) in MES buffer. (DOCX 211 kb) [file 12866_2017_1140_MOESM2_ESM.docx]

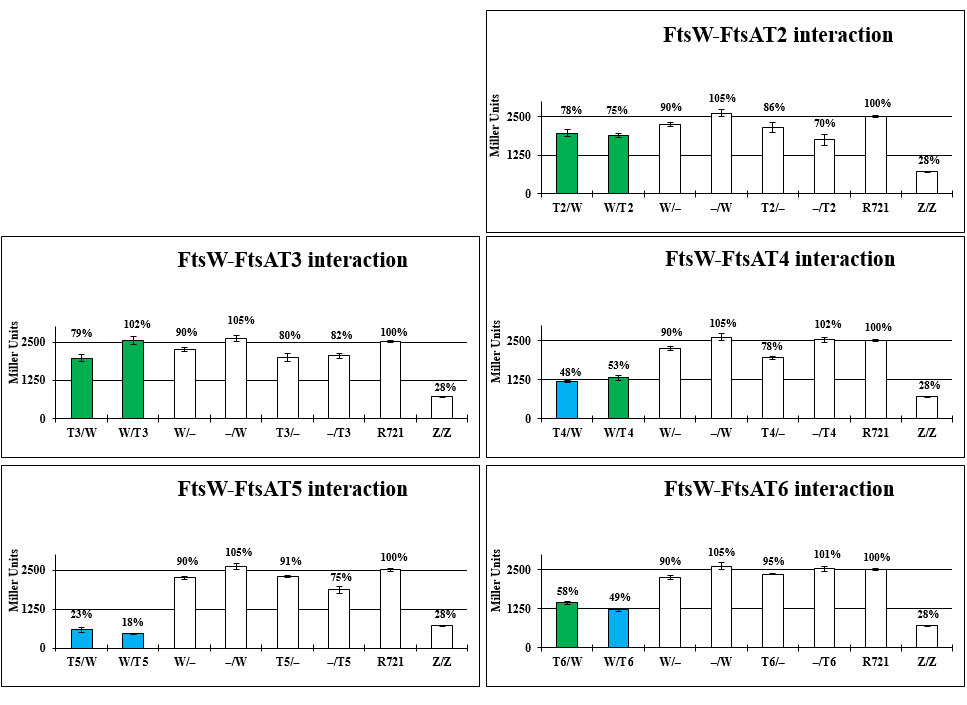
**Fig S3**

Supplement: Supplementary file 3 — Interactions between FtsANg truncations (T2, T3, T4, T5 and T6) and FtsWNg (W) by B2H assay. Values of less than 50% (<1250 Miller Units) indicate a positive interaction between two proteins (blue bars) while values of more than 50% (>1250 Miller Units) indicate a negative interaction (green bars). (DOCX 66 kb) [file 12866_2017_1140_MOESM3_ESM.docx]
